# Supplementary material for: Allosteric regulation and crystallographic fragment screening of SARS-CoV-2 NSP15 endoribonuclease
Source: Nucleic Acids Res. 2023 Apr 28;51(10):5255–70. doi: 10.1093/nar/gkad314 (PMC10250223; doi:10.1093/nar/gkad314)
Supplement: gkad314_Supplemental_Files [file gkad314_supplemental_files.zip › Supplementary datasets.zip!/183/Refine_4/01-BUSTER/Cycle-4/shell.01/R_Rfree.html]

# Rxpct factors in resolution bins at cycle 0


|  |  |  |  |  |  |  |  |  |
| --- | --- | --- | --- | --- | --- | --- | --- | --- |
|  | **<d> (Å)**  Rxpct\_Work | Rxpct\_Free | Rxpct\_All | |  |  |  |  |
|  | 14.0455 | 0.3025 | 0.3420 | 0.3041 |  |
|  | 8.1092 | 0.2221 | 0.2648 | 0.2246 |  |
|  | 6.2814 | 0.2442 | 0.2556 | 0.2448 |  |
|  | 5.3087 | 0.2220 | 0.2611 | 0.2238 |  |
|  | 4.6818 | 0.1951 | 0.2130 | 0.1961 |  |
|  | 4.2349 | 0.2027 | 0.1872 | 0.2019 |  |
|  | 3.8955 | 0.2026 | 0.2241 | 0.2036 |  |
|  | 3.6265 | 0.2157 | 0.2643 | 0.2182 |  |
|  | 3.4065 | 0.2101 | 0.2295 | 0.2112 |  |
|  | 3.2223 | 0.2031 | 0.2147 | 0.2037 |  |
|  | 3.0650 | 0.1973 | 0.1886 | 0.1969 |  |
|  | 2.9287 | 0.1909 | 0.1871 | 0.1908 |  |
|  | 2.8091 | 0.1917 | 0.1993 | 0.1921 |  |
|  | 2.7031 | 0.1941 | 0.2110 | 0.1950 |  |
|  | 2.6082 | 0.2020 | 0.2202 | 0.2030 |  |
|  | 2.5227 | 0.1978 | 0.2151 | 0.1988 |  |
|  | 2.4450 | 0.1988 | 0.2188 | 0.1998 |  |
|  | 2.3741 | 0.2128 | 0.2185 | 0.2131 |  |
|  | 2.3091 | 0.2334 | 0.2476 | 0.2340 |  |
|  | 2.2491 | 0.3031 | 0.3117 | 0.3034 |  |
|  | **Overall** | 0.2129 | 0.2292 | 0.2137 |  |

# Rxpct factors in resolution bins at cycle 1


|  |  |  |  |  |  |  |  |  |
| --- | --- | --- | --- | --- | --- | --- | --- | --- |
|  | **<d> (Å)**  Rxpct\_Work | Rxpct\_Free | Rxpct\_All | |  |  |  |  |
|  | 14.0455 | 0.3027 | 0.3423 | 0.3043 |  |
|  | 8.1092 | 0.2220 | 0.2649 | 0.2246 |  |
|  | 6.2814 | 0.2438 | 0.2554 | 0.2444 |  |
|  | 5.3087 | 0.2215 | 0.2611 | 0.2233 |  |
|  | 4.6818 | 0.1946 | 0.2131 | 0.1956 |  |
|  | 4.2349 | 0.2020 | 0.1868 | 0.2012 |  |
|  | 3.8955 | 0.2017 | 0.2238 | 0.2028 |  |
|  | 3.6265 | 0.2147 | 0.2636 | 0.2172 |  |
|  | 3.4065 | 0.2090 | 0.2286 | 0.2101 |  |
|  | 3.2223 | 0.2021 | 0.2140 | 0.2027 |  |
|  | 3.0650 | 0.1965 | 0.1886 | 0.1961 |  |
|  | 2.9287 | 0.1905 | 0.1873 | 0.1904 |  |
|  | 2.8091 | 0.1915 | 0.1997 | 0.1919 |  |
|  | 2.7031 | 0.1940 | 0.2116 | 0.1950 |  |
|  | 2.6082 | 0.2021 | 0.2204 | 0.2031 |  |
|  | 2.5227 | 0.1978 | 0.2150 | 0.1988 |  |
|  | 2.4450 | 0.1988 | 0.2186 | 0.1997 |  |
|  | 2.3741 | 0.2126 | 0.2188 | 0.2129 |  |
|  | 2.3091 | 0.2330 | 0.2482 | 0.2337 |  |
|  | 2.2491 | 0.3034 | 0.3118 | 0.3037 |  |
|  | **Overall** | 0.2124 | 0.2291 | 0.2132 |  |

# Rxpct factors in resolution bins at cycle 2


|  |  |  |  |  |  |  |  |  |
| --- | --- | --- | --- | --- | --- | --- | --- | --- |
|  | **<d> (Å)**  Rxpct\_Work | Rxpct\_Free | Rxpct\_All | |  |  |  |  |
|  | 14.0455 | 0.3028 | 0.3425 | 0.3044 |  |
|  | 8.1092 | 0.2219 | 0.2650 | 0.2245 |  |
|  | 6.2814 | 0.2433 | 0.2550 | 0.2438 |  |
|  | 5.3087 | 0.2207 | 0.2610 | 0.2226 |  |
|  | 4.6818 | 0.1940 | 0.2132 | 0.1951 |  |
|  | 4.2349 | 0.2011 | 0.1866 | 0.2004 |  |
|  | 3.8955 | 0.2008 | 0.2236 | 0.2019 |  |
|  | 3.6265 | 0.2136 | 0.2631 | 0.2162 |  |
|  | 3.4065 | 0.2081 | 0.2278 | 0.2091 |  |
|  | 3.2223 | 0.2012 | 0.2135 | 0.2019 |  |
|  | 3.0650 | 0.1958 | 0.1888 | 0.1955 |  |
|  | 2.9287 | 0.1902 | 0.1876 | 0.1900 |  |
|  | 2.8091 | 0.1913 | 0.2000 | 0.1917 |  |
|  | 2.7031 | 0.1940 | 0.2119 | 0.1950 |  |
|  | 2.6082 | 0.2022 | 0.2208 | 0.2032 |  |
|  | 2.5227 | 0.1979 | 0.2153 | 0.1989 |  |
|  | 2.4450 | 0.1989 | 0.2186 | 0.1999 |  |
|  | 2.3741 | 0.2125 | 0.2190 | 0.2128 |  |
|  | 2.3091 | 0.2330 | 0.2486 | 0.2336 |  |
|  | 2.2491 | 0.3038 | 0.3120 | 0.3041 |  |
|  | **Overall** | 0.2119 | 0.2290 | 0.2127 |  |

# Rxpct factors in resolution bins at cycle 3


|  |  |  |  |  |  |  |  |  |
| --- | --- | --- | --- | --- | --- | --- | --- | --- |
|  | **<d> (Å)**  Rxpct\_Work | Rxpct\_Free | Rxpct\_All | |  |  |  |  |
|  | 14.0455 | 0.3085 | 0.3461 | 0.3100 |  |
|  | 8.1092 | 0.2183 | 0.2706 | 0.2214 |  |
|  | 6.2814 | 0.2254 | 0.2479 | 0.2266 |  |
|  | 5.3087 | 0.1964 | 0.2301 | 0.1979 |  |
|  | 4.6818 | 0.1744 | 0.1994 | 0.1757 |  |
|  | 4.2349 | 0.1764 | 0.1814 | 0.1767 |  |
|  | 3.8955 | 0.1780 | 0.2160 | 0.1798 |  |
|  | 3.6265 | 0.1896 | 0.2463 | 0.1925 |  |
|  | 3.4065 | 0.1857 | 0.2038 | 0.1866 |  |
|  | 3.2223 | 0.1848 | 0.2116 | 0.1863 |  |
|  | 3.0650 | 0.1834 | 0.1931 | 0.1839 |  |
|  | 2.9287 | 0.1832 | 0.2038 | 0.1841 |  |
|  | 2.8091 | 0.1858 | 0.2098 | 0.1869 |  |
|  | 2.7031 | 0.1918 | 0.2136 | 0.1930 |  |
|  | 2.6082 | 0.2035 | 0.2212 | 0.2045 |  |
|  | 2.5227 | 0.2030 | 0.2279 | 0.2045 |  |
|  | 2.4450 | 0.2062 | 0.2292 | 0.2073 |  |
|  | 2.3741 | 0.2196 | 0.2294 | 0.2200 |  |
|  | 2.3091 | 0.2429 | 0.2728 | 0.2442 |  |
|  | 2.2491 | 0.3071 | 0.3120 | 0.3073 |  |
|  | **Overall** | 0.1988 | 0.2243 | 0.2001 |  |
